# Using hair cortisol analysis to understand the biological factors that affect black-footed ferret (*Mustela nigripes*) stress physiology

**DOI:** 10.1093/conphys/coab033

**Published:** 2021-05-11

**Authors:** R M Santymire, N Ali, P E Marinari, T M Livieri

**Affiliations:** 1 Conservation and Science Department, Lincoln Park Zoo, 2001 N. Clark St, Chicago, IL 60614, USA; 2 Committee on Evolutionary Biology, University of Chicago, 1025 E. 57th St, Chicago, IL 60637, USA; 3 Center for Species Survival, Smithsonian Conservation Biology Institute, 1500 Remount Rd, Front Royal, VA 22630, USA; 4 Prairie Wildlife Research, PO Box 308, Wellington, CO 80549, USA

**Keywords:** Age, enzyme immunoassay, *ex situ*, glucocorticoids, season, sex, wild

## Abstract

The black-footed ferret (*Mustela nigripes*) was driven to near extinction due to habitat loss and an introduced disease, sylvatic plague (*Yersinia pestis*). After 35 years of breeding in *ex situ* facilities, the black-footed ferret has been experiencing infertility with seminal traits declining in males and only about a third of breeding-aged females are whelping. Our goal was to use hair cortisol analysis to determine if the *ex situ* population was experiencing chronic stress that was affecting reproduction by comparing captive ferrets to wild individuals. Our specific objectives were to (i) compare hair cortisol concentrations (HCCs) between age classes (juveniles versus adults), (ii) compare the HCCs of *in situ* and across different *ex situ* facilities and (iii) determine the relationship between HCCs and reproductive success. Overall, wild juveniles had higher HCC than wild adults. Our generalized linear mixed model determined that the parameters that best predict HCC for adults were the interactions among sex, *in situ* versus *ex situ* facilities and season. During both seasons, wild females had higher HCCs compared to the *ex situ* females. During the breeding season, male HCCs across breeding facilities varied and males at the breeding facility with the largest ferret habitats had HCCs similar to wild males. At one breeding facility, HCC was higher in males that sired compared to those that did not sire. In conclusion, *ex situ* ferrets do not have higher HCC than wild individuals when controlling for season and *ex situ* habitat size, and *ex situ* males with higher HCC tended to sire. This suggests that HCC may be metabolically driven and/or that low HCC may be an indication of hypothalamus–pituitary–adrenal axis dysregulation and warrants further investigation both for laboratory validation and biological relevance.

## Teaser

For the first time, cortisol was extracted from hair and used to compare glucocorticoid production among wild and captive black-footed ferrets. Several biological factors influenced hair cortisol concentrations including season, sex, age and location. These unique findings will assist with determining the etiology of infertility in the black-footed ferret.

## Introduction

Anthropogenic activities have put pressure on wildlife habitat and populations resulting in nearly one million species threatened with extinction (Brondizio *et al*., 2019). There are many strategies employed to offset these pressures; however, often times to prevent extinction an *ex situ* population needs to be established. This was the case for the black-footed ferret (*Mustela nigripes*), North America’s only endemic ferret species. This species was driven to near extinction due to habitat loss, disease and a decline in its main prey species, the prairie dog (*Cynomys* spp.). In an effort to prevent extinction, the last 24 black-footed ferrets were removed from the wild near Meeteetse, WY, from 1985 to 1987. Of those, only 15 produced offspring that survived with seven considered to be genetic founders of the population (Graves *et al*., 2019). After nearly 35 years of captive breeding, which is overseen by the US Fish and Wildlife Service (USFWS), more than 9600 ferrets have been produced (Marinari, 2019). And since 1991, captive-born black-footed ferrets have been pre-conditioned and reintroduced into the wild throughout North America’s Great Plains (Jachowski and Lockhart, 2009). However, managing wildlife *ex situ* can be challenging because of the difficulty to completely simulate their natural habitat including both environmental and social conditions. Therefore, zoos and other breeding facilities design habitats that meet as many of the of the animals’ needs as possible using species natural history while striving to maximize reproductive success.

Facilities accredited by the Association of Zoos and Aquariums strive to maintain the genetic health of the more than 500 species in their care for the next 100 years without acquiring new individuals from the wild. However, this can be challenging especially when only 20% of the breeding recommendations are successful across all Species Survival Plans^®^ (Faust *et al*., 2019). For the black-footed ferret, there are currently no novel genes to bring into the population naturally, but over time the black-footed ferret *ex situ* has maintained nearly 86% of the population’s gene diversity (Graves *et al*., 2019). However, we have observed signs of inbreeding depression, which is the result of increased mating between closely related individuals (Wright, 1977; Charlesworth and Charlesworth, 1987; Thornhill, 1993; Frankham, 2005). Species experiencing inbreeding depression are at a high risk for fertility issues, such as sperm degradation (Fitzpatrick and Evans, 2009; Brekke *et al*., 2010), reduced pregnancy rates (van Noordwijk and Scharloo, 1981; González-Recio *et al*., 2007) and negative impacts on sperm quality, including motility, concentration and morphology (Fitzpatrick and Evans, 2009). Carnivorans are known to be susceptible to inbreeding depression with case studies in African lions in the Ngorongoro Crater in Tanzania (Wildt *et al*., 1987; Munson *et al*., 1996) and the Florida panther (Wildt *et al*., 1994).

For the black-footed ferret, Santymire *et al*. (2019) examined a 20-year interval of captive breeding to investigate the effects of increasing inbreeding coefficient (*F*) on reproductive traits and found that as *F* increased, the percentage of normal spermatozoa in the ejaculate declined. Specifically, we have observed a decrease in the percentage of normal spermatozoa in ejaculates, which started at 50% in 1990 and declined to 25% by 2000 and remained consistent through 2007 (Santymire *et al*., 2019) and through 2019 (Per Comm. R.M. Santymire). For female reproductive success, pregnancy rates declined from 60% in the 1990s to 38% in 2019 (Marinari, 2019). However, wild black-footed ferrets, who are descendants of the *ex situ* population, have a higher percentage of normal sperm (45–55% normal sperm; Santymire *et al*., 2006). Further, an estimated 75% or more of wild females observed are associated with a litter in the summer and fall (Pers. comm: Travis M. Livieri). Therefore, *ex situ* black-footed ferrets could be experiencing environmental-dependent inbreeding depression (EDID), where the magnitude of inbreeding depression is enhanced by environmental conditions, possibly mediated/influenced by the neuroendocrine stress response (Cheptou and Donohue, 2011).

Stress caused by the *ex situ* environment may be due to a number of factors, such as lack of exercise, range allowance, light conditions, proximity of conspecifics, sounds, odours, temperature and tactile experiences (Morgan and Tromborg, 2007). Research has shown that chronic stress induced by the *ex situ* environment can put individuals at risk of reduced health including shorter lifespans, abnormal estrous cycling and high infant mortality in elephants (Clubb *et al*., 2008; Mason and Veasey, 2010). High prevalence of disease, premature death and reproductive issues were also observed in *ex situ* cheetahs Acinonyx jubatus (Terio *et al*., 2004). Additionally, evidence has shown that *ex situ* cheetahs have elevated glucocorticoids (GCs), which are steroidal hormones released through the activation of the hypothalamic–pituitary–adrenal (HPA) axis (Reeder and Kramer, 2005), indicating chronic stress as a major contributor to their difficulty living *ex situ* (Terio *et al*., 2004). The abnormal production of GCs can result in the down regulation of all non-essential processes, including reproduction. Specifically, elevated GC concentrations can directly inhibit release of gonadotropin-releasing hormone, which can then reduce the synthesis and release of follicle-stimulating hormone and luteinizing hormone, thus, reducing the gonadal hormone production including testosterone in males and progesterone and estrogen in females (Whirledge and Cidlowski, 2017). However, the main function of GCs is to mobilize energy through increased gluconeogenesis, and they are affected by several biological factors including age, sex, nutritional state, circadian rhythm, circannual rhythm and thermoregulation (reviewed in Reeder and Kramer, 2005).

GC production analysis has traditionally been measured via cortisol or corticosterone in blood, faeces, urine and/or saliva, which reflect acute responses to a perceived stressor. Recent endocrinological research has used hair samples for GC analysis (Stalder and Kirschbaum, 2012). The advantages of using hair over traditional biomaterials include that samples can be stored at room temperature because the steroids deposited in the hair will not degrade under variable environmental conditions and a longer term perspective of GC production (weeks to months; Stalder and Kirschbaum, 2012). The mechanism by which hormones are deposited into hair is not fully understood, but it is likely that GCs diffuse across the plasma membranes from blood capillaries into the growing hair cell (Stalder and Kirschbaum, 2012) and remain in the shaft during the rest phase (Harkey, 1993; Henderson, 1993; Thieme *et al*., 2003). However, steroids may be produced locally within the hair follicle (Ito *et al*., 2005; Keckeis *et al*., 2012); therefore, hormones measured in hair may be a combination of long-term steroids stored during growth and recent steroid production from the hair follicle or surrounding glands (reviewed in Kalliokoski *et al*., 2019; Koren *et al*., 2019). Additionally, repeated Adrenocorticotrophic Hormone (ACTH) challenges have resulted in increases of hair cortisol concentrations (HCCs), thus demonstrating that hair does reflect integrated levels of circulating GCs (Endo *et al*., 2018).

Overall, hair steroid analysis can be effective at comparing GC production across populations if certain precautions are taken, including collecting hair from the same location across individuals at the same time of year since hair growth rate can vary across individuals, or within an individual across seasons and along body regions (Carlitz *et al*., 2015; Fourie *et al*., 2016). Here, our goal was to use hair cortisol analysis to determine if the *ex situ* population of black-footed ferrets was experiencing chronic stress that was leading to abnormal concentrations of GCs that were affecting reproduction and to compare results to those of a wild population. Previous research has demonstrated that the *ex situ* population of black-footed ferrets (*n* = 72) produced higher faecal glucocorticoid metabolites (FGMs) than wild ferrets (*n* = 5; Poessel *et al*., 2011). However, the wild samples were collected in a different season compared to the *ex situ* samples. Additionally, these populations eat differently, both in respect to food items, horsemeat diet for *ex situ* and prairie dogs for wild, frequency (daily for *ex situ* and unknown for the wild) and quantity, which can affect defecation rates and then faecal hormone metabolite analysis (Goymann, 2012; Palme, 2019). Our specific objectives were to (i) compare HCCs between age classes (juveniles versus adults), (ii) compare the adult black-footed ferret HCCs of *in situ* and *ex situ* populations as well as the HCCs across different breeding facilities to determine if *ex situ* black-footed ferrets were experiencing higher levels of stress than they would naturally and (iii) determine the relationship between HCCs and reproductive success. We predicted that juveniles would have higher HCC than adults as observed in other species (Pryce *et al*., 2002; Fourie and Bernstein, 2011) and that *ex situ* black-footed ferrets would have higher HCC than *in situ* individuals.

## Materials and methods

### Study animals

As obligate predators of the prairie dog, wild black-footed ferret populations are established only within the confines of prairie dog colonies (Biggins *et al*., 2006) and utilize prairie dog burrows. Black-footed ferrets are a polygynous species with a strict breeding season in the spring (February through May) as the photoperiod begins to lengthen. With the onset of spring, spermatogenesis is initiated in males, as is an increase in testosterone production, causing the testes to enlarge (Hillman and Carpenter, 1984; Carvalho *et al*., 1991; Williams *et al*., 1991; Brown, 1997). Male testes firmness is an indication of breeding readiness (Hillman *et al*., 1984; Carvalho *et al*., 1991; Williams *et al*., 1991). Males annually defend larger territories around smaller female territories (Livieri and Anderson, 2012; Eads *et al.*, 2014). However, in the *ex situ* breeding program, males are paired with females based on a pedigree analysis and not on mate choice selection (Graves *et al*., 2019). After a 42–43 day gestation period, parturition of altricial kits occurs during May–June. Maternal care is intensive through late summer. In the wild, kits reach adult size in the early fall and disperse from the natal area (Biggins and Eads, 2017).

For the *ex situ* populations of black-footed ferrets used in this study (*n* = 126 males and 33 females; 700 g to 1000 g; < 1 to 4 years old), we sampled when individuals were anesthetized for either management sampling or physical examinations. These black-footed ferrets were housed individually at the National Black-footed Ferret Conservation Center (FCC; Carr, CO; *n* = 121, males = 99, females = 22), Smithsonian Conservation Biology Institute (SCBI; Front Royal, VA; *n* = 24, males = 13, females = 11) or Louisville Zoological Gardens (LZG; Louisville, KY, *n* = 14, males = 14, females = 0). At FCC and LZG, ferrets were housed in indoor cages (1.0 m × 1.3 m × 1.0 m) with upper and lower nestboxes filled with Alpha-dri^®^ substrate (Shepherd Speciality Paper, Watertown, TN, USA). At SCBI, ferrets were housed in indoor enclosures (3.6 m × 6.0 m × 4.0 m) with a mulch substrate and contained nestboxes filled with Alpha-dri^®^ substrate. At all facilities, lighting was both natural (provided by skylights) and artificial (via fluorescent or LED illumination; set to the natural photoperiod). Black-footed ferrets were fed 75 to 100 g of a commercially available horsemeat diet (TOR; product was handled according to manufacturer’s recommendations) or rodent carcass daily and were provided with *ad libitum* water.

Wild black-footed ferrets (*n* = 186; males = 107, females = 79) were all wild born and were trapped from the fall of 2014 through the spring of 2017 during monitoring surveys at the Conata Basin/Badlands National Park reintroduction site. The Conata Basin is a portion of the Buffalo Gap National Grasslands (43°46 N and 102°14 W), administered by the United States Forest Service, in southwestern South Dakota, USA, and is directly adjacent to Badlands National Park. These two federal land units encompass 58 222 ha of mixed grass prairie with more than 5340 ha of black-tailed prairie dog colonies (*Cynomys ludovicianus*). Black-footed ferret reintroduction began in 1994 and ended in 1999. Sixty or more litters were documented annually from 2000 to 2008 and the site is considered self-sustaining (Jachowski and Lockhart, 2009) but requires intensive disease management, vector control in prairie dog burrows and direct vaccination of black-footed ferrets, to reduce the incidence of sylvatic plague (*Yersinia pestis*). Trapping and immobilization followed protocols of the Black-Footed Ferret Recovery Implementation Team (Kreeger, 1998; Black-footed Ferret Recovery Team, 2016). Briefly, animals were cage-trapped at night and returned to the same location following examination and recovery from anesthesia, usually within 1 hr of capture. All trapping was authorized by the Black-Footed Ferret Recovery Implementation Team under permit #TE064682-1 and was conducted by the US Forest Service, National Park Service and Prairie Wildlife Research as part of routine population monitoring for Conata Basin. All animal experiments conformed to the Guide for Care and Use of Laboratory Animals and were approved by the Lincoln Park Zoo Research Committee (Chicago, IL) and USFWS (Carr, CO).

### Sample collection and processing

Hair was collected during medical examinations while black-footed ferrets were anesthetized in an anesthesia induction chamber via inhalation of isoflurane (Kreeger, 1998). All hair samples were shaved from the ventral side of the neck, under the chin about 1 inch by 1 inch. This area was chosen because the individual could not reach it for licking/grooming, which could add salivary hormones onto the hair, thereby confounding our cortisol measurements. This was also the area that was shaved for blood collection via the jugular vein. All sample collection took place either during the breeding season (March and April) or during routine medical examinations (December and January). Shaved hair was placed in pre-labelled, individual opaque paper envelopes and stored at room temperature until analysis.

Each hair sample was washed with 5.0 ml of 90% MeOH (methanol:distilled water) and placed on a mixer (Glas-Col, Terre Haute, IN) for 1 min at setting 50 to ensure consistent mixing across all samples. The methanol was decanted and an additional 5.0 ml of MeOH was added to the hair. This process was repeated three times. Hair samples were then placed in individual plastic trays until completely dry (~3 days; Schell *et al*., 2017).

### Hormone extraction

Dried hair was pulverized to a fine powder (Omni Bead Ruptor 24, Kennesaw, GA, USA; on settings: 6.8 m/s, four 50 s intervals with 15 s break). Pulverized hair weighing 0.02 ± 0.005 g was then placed into pre-labelled plastic tubes and combined with 2 ml of 90% MeOH, vortexed briefly and agitated on the Glas-col mixer for 4 hr at speed 50. Tubes were then centrifuged (Sorvall R-4, Thermo-Scientific, Waltham, MA) for 15 min at 500 × *g* at 10°C, and the supernatant was decanted into clean, pre-labelled plastic tubes. The supernatant was then evaporated in a hot-water bath (DC30; Fisher Scientific, Waltham, MA, USA; 60°C) with air blowers in each individual tube. For assay analysis, dried extract was reconstituted with 250 μl of assay buffer (0.2 M NaH_2_PO_4_, 0.2 M Na_2_HPO_4_, NaCl, pH 7.0) to produce a four times concentrated sample. Glass beads were added to each tube and vortexed briefly. Samples were then sonicated (FS220, Fisher Scientific) for 20 min before analysis (Schell *et al*., 2017).

### Cortisol enzyme immunoassay

We measured HCC by modifying a previously described cortisol enzyme immunoassay (EIA; Loeding *et al*., 2011). Briefly, 96-well Nunc plates (Thermo-Scientific) were coated with a goat anti-rabbit antibody (1:1000, Arbor Assays, Ann Arbor, MI, USA) and incubated at room temperature for 1 day. Contents were poured out and a blocking buffer (Arbor Assays) was added and incubated at room temperature for 1 day. The contents were poured off and then the plates were dried and stored at 5°C until used. On the day of the EIA analysis, polyclonal cortisol antiserum (R4866) and cortisol-3-CMO-peroxidase (provided by C. Munro (University of California, Davis, California) were used at dilutions of 1:375000 and 1:200000, respectively, and then used in the EIA protocol described by Munro and Stabenfeldt (1984). The cross-reactivities for cortisol antiserum were the following: cortisol, 100%; prednisolone, 9.9%; prednisone, 6.3%; cortisone, 5%; corticosterone, 0.7%; deoxycorticosterone, 0.3%; 21-deoxycortisone, 0.5%; 11-deoxycortisol, 0.2%; progesterone, 0.2%; 17α-hydroxyprogesterone, 0.2%; pregnenolone, 17α-hydroxypregnenolone, anderostenedione, testosterone, androsterone, dehydroepiandrosterone, dehydroisoandrosterone-3-sulfate, aldosterone, estradiol-17β, estrone, estriol, spironolactone and cholesterol, 0.1% (Young *et al*., 2004; Loeding *et al*., 2011).

To biochemically validate the cortisol EIA (Möstl and Palme, 2005), we evaluated the parallel relationship, using the Spearman rank-order test, between binding inhibition curves of hair extract dilutions (neat, twice concentration and quadrupled concentration) and the cortisol standard (*r* = 0.999), and the significant recovery (88%) of exogenous cortisol (1.95–1000 pg/well) added to hair extracts (*y* = 0.955x − 0.913; *R*^2^ = 0.999) using linear regression analysis. Assay sensitivity was 1.95 pg/well and the intra- and inter-assay coefficients of variation were <15%.

### Statistical analysis

Our overarching goal was to build a generalized linear mixed model (GLMM) that explains how various factors (season, sex and *ex situ* versus *in situ*) influence black-footed ferret HCCs. We first evaluate the normality of our dataset through visual inspection of histogram, quantile plots and the Cullen and Grey graph using the fitdistrplus package (Delignette-Muller and Dutang, 2015), which uses Pearson’s distribution to determine what data transformation to perform, which was the log-transformation. However, our dataset did not meet the homogeneity of variance assumption based on the Fligner–Killeen test of homogeneity of variances from the ‘onewaytests’ package (Dag *et al*., 2018) Therefore, we used Welch’s *T*-test from the ‘onewaytests’ package for pairwise comparisons, which accommodates normal data (our log-transformed HCC data) with non-homogeneous variances. The pairwise tests allowed us to further investigate the relationships established by our GLMM.

We used our log-transformed data in a GLMM to determine what factors may influence HCC in the black-footed ferret. Sex, *ex situ* versus *in situ*, season and their interactions were evaluated. Sample year, individual identity and locations (Conata Basin, SD for wild, SCBI, FCC and LZG) were used as random effects. This model was fit in RStudio (Version 1.1.412; RStudio Team, 2020) using the packages ‘lmerTest’ and ‘MuMIn’ (Kuznetsova *et al*., 2017; Bartoń, 2018). All figures show untransformed HCC results. All analyses were conducted in RStudio (Version 1.1.412) using the following packages: ggplot2, dplyr and onewaytests (Wickham, 2016; Dag *et al*., 2018; Wickham *et al*., 2019).

To determine the relationship between HCC and reproductive success, we used reproductive records compiled from each *ex situ* facility and matched them for years in which we had HCC data (FCC, SCBI and LZG where available). Groups were compared using Welch’s *T*-test in RStudio, as above.

## Results

### Age

**Figure 1:**
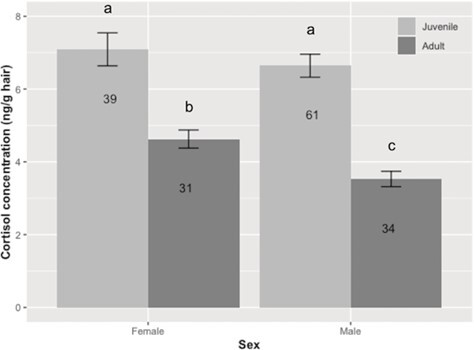
Mean (±SEM) HCCs (ng/g) of wild black-footed ferrets during the non-breeding season. Letters indicate differences (*P* < 0.05) among age class and sex. Number in the bar is the number of individuals evaluated.

No kits were available in our dataset for the breeding season, so all inferences regarding comparisons of age classes are for the non-breeding season. Overall, wild juveniles had higher (*F*_1, 139.16_ = 84.56, *P* < 0.0001) HCC than wild adults during the non-breeding season (Fig. 1). Juvenile females had higher HCC than adult females (*F*_1, 67.28_ = 25.35, *P* < 0.0001) and adult males (*F*_1, 70.70_ = 63.56, *P* < 0.0001; Fig. 1). Juvenile males had higher HCC than adult males (*F*_1, 66.16_ = 71.79, *P* < 0.0001) and adult females (*F*_1, 73.62_ = 27.07, *P* < 0.0001). Juvenile male and female HCC were similar (*F*_1, 74.44_ = 0.50, *P* > 0.05); however, adult females had higher (*F*_1, 61.27_ = 14.15, *P* < 0.005) HCC than adult males during the non-breeding season. Because age affected HCCs, we then compared adult HCC between *in situ* and *ex situ* populations.

### Explanatory model

The parameters that best predicted HCC for adults were the interactions among sex, *in situ* versus *ex situ* and season; all predictors were found to be significant in the model except *in situ* versus *ex situ* (Table 1). The marginal *R*^2^ (fixed effects only) for the model is 0.23 while the conditional *R*^2^ (fixed and random effects combined) for the model is 0.82. This implies that the random effects (individual ID, sample year, location) explain a large proportion of the variance. Sample year explains 36.1% of the variation not explained by fixed effects, individual identity accounts for 23.6% and location accounts for 16.8% (Table 1).

**Table 1:** Model parameters from the fitted general linear mixed effects model for black-footed ferret hair cortisol concentrations (HCCs)

Fixed effects	Value	Standard error	DF	*t*-value	*P*-value
(Intercept)	1.24	0.22	14.79	5.67	**<0.0001**
*In situ* versus *ex situ*	0.39	0.21	7.54	1.82	0.11
Sex (M versus F)	0.26	0.11	209.82	2.40	**<0.05**
Season (breeding versus non-breeding)	−0.62	0.15	225.04	−4.19	**<0.0001**
Sex * season	−0.72	0.14	217.40	−4.97	**<0.0001**
*In situ* versus *ex situ* * season	0.62	0.16	232.40	3.79	**<0.0005**
					
**Random effects**	**Standard deviation**	**% Variance explained**			
Animal ID	0.26	23.6%			
Location	0.22	16.8%			
Sample year	0.33	36.1%			
Residual	0.26	23.4%			

*Ex situ* populations compared to *in situ* population, comparing males (M) and females (F) across seasons (breeding versus non-breeding)

### Effect of *ex situ* versus *in situ*, sex and season on HCC

**Figure 2:**
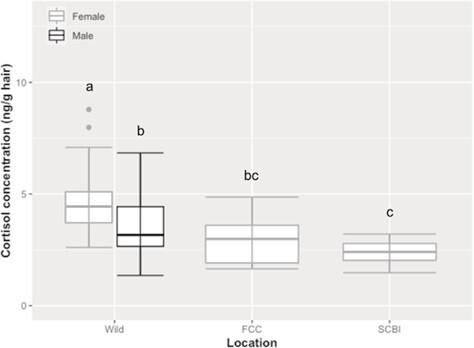
Boxplot of median cortisol concentrations (HCC; ng/g hair) of adult male and female black-footed ferrets by location including the wild individuals (*n* = 31 females, *n* = 34 males) from Conata Basin and from *ex situ* facilities including FCC (*n* = 10 females) and the SCBI (*n* = 11 females) during the non-breeding season. The median value is the center line in each box. The upper whisker extends from Q3 to the largest value and does not extend further than 1.5 * IQR; the lower whisker extends from Q1 to the smallest value and does not extend beyond 1.5 * IQR. Any data points that fall beyond the upper and lower whiskers are depicted as outliers, which were included in our analyses. Letters indicate differences (*P* < 0.05) across location and sex.

While our GLMM did not find *ex situ* versus *in situ* alone to be significant in determining HCC, the interaction of *ex situ* versus *in situ* and season was significant. Specifically, during the non-breeding season, wild adult females had the highest HCC compared to wild adult males (*F*_1, 61.27_ = 14.15, *P* < 0.005), FCC females (*F*_1, 11.85_ = 12.25, *P* < 0.05) and SCBI females (*F*_1, 18.52_ = 49.92, *P* < 0.0001; Fig. 2). Wild male HCC was similar to FCC females (*F*_1, 13.51_ = 1.64, *P* > 0.10) but was higher than SCBI females (*F*_1, 23.37_ = 12.94, *P* < 0.005; Fig. 2). Adult female HCCs from both captive locations were similar (*F*_1, 15.19_ = 1.42, *P* > 0.10).

**Figure 3:**
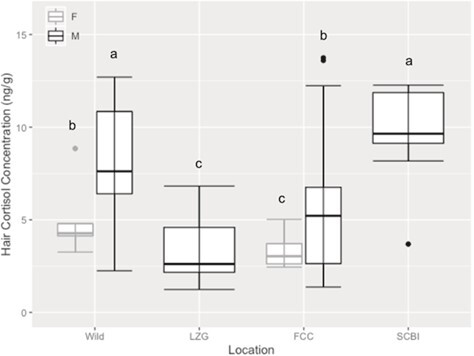
Boxplot of median HCCs (ng/g) of adult male and females black-footed ferret by location including the wild individuals (*n* = 9 females, *n* = 12 males) from Conata Basin and from *ex situ* facilities including FCC (*n* = 14 females; *n* = 97 males), the SCBI (*n* = 13 males) and the LZG (*n* = 14 males) during the breeding season. The median value is the center line in each box. The upper whisker extends from Q3 to the largest value and does not extend further than 1.5 * IQR; the lower whisker extends from Q1 to the smallest value and does not extend beyond 1.5 * IQR. Any data points that fall beyond the upper and lower whiskers are depicted as outliers, which were included in our analyses. Letters indicate differences (*P* < 0.05) across location and sex.

During the breeding season, wild males and SCBI males had similar (*F*_1, 22.90_ = 0.28, *P* > 0.10) HCC but had higher HCC than wild females (wild: *F*_1, 17.80_ = 7.25, *P* < 0.05; SCBI: *F*_1, 19.23_ = 11.30, *P* < 0.001), LZG males (wild: *F*_1, 21.58_ = 32.91, *P* < 0.0005; SCBI: *F*_1,23.30_ = 40.99, *P* < 0.0005), FCC females (wild: *F*_1, 17.38_ = 20.60, *P* < 0.005; SCBI: *F*_1, 18.69_ = 27.38, *P* < 0.0005) and FCC males (wild: *F*_1, 14.37_ = 7.00, *P* < 0.05; SCBI: *F*_1, 15.85_ = 11.53, *P* < 0.001; Fig. 3). LZG males had lower HCC than FCC males (*F*_1, 19.86_ = 26.32, *P* < 0.0005) and wild females (*F*_1, 20.93_ = 16.21, *P* < 0.005) but similar to FCC females (*F*_1, 19.65_ = 3.32, *P* > 0.05). Adult wild females had higher (*F*_1, 14.91_ = 5.84, *P* < 0.05, *P* = 0.029) HCC than FCC females during the breeding season but were similar (*F*_1, 14.41_ = 0.25, *P* > 0.05, *P* = 0.63) to FCC males (Fig. 3).

HCC for FCC females was similar (*F*_1, 15.69_ = 0.94, *P* > 0.1) across the seasons. HCC also was similar (*F*_1, 12.57_ = 0.00, *P* > 0.1) across the seasons for wild females. In contrast, HCC for wild males was higher (*F*_1, 14.92_ = 23.49, *P* < 0.005) during the breeding season compared to the non-breeding season. FCC, SCBI and LZG male data were only available during the breeding season so could not be compared across season.

### Reproductive outcomes

When determining the relationship between HCCs and reproductive success, we found that HCC from sires at FCC (*n* = 51; 6.43 ± 0.36 ng/g) had higher (*F*_1, 105.02_ = 4.66, *P* = 0.033) HCC than males who did not sire (*n* = 94; 5.38 ± 0.29 ng/g). This was not the case for SCBI or LZG males. SCBI sires (*n* = 11; 4.59 ± 0.98 ng/g) had similar (*F*_1, 5.92_ = 1.82, *P* > 0.10) HCC to males who did not sire (*n* = 5; 7.66 ± 1.94 ng/g). Likewise, HCC for males who sired (*n* = 6; 3.41 ± 0.90 ng/g) was similar (*F*_1, 7.05_ = 0.09, *P* > 0.10) to males who did not sire (*n* = 8; 2.68 ± 0.43 ng/g) at LZG. For females, we found that HCC for females who whelped (*n* = 4; 2.30 ± 0.31 ng/g) was similar (*F*_1,5.33_ 0.14, *P* > 0.10) to females who did not whelp (*n* = 4; 2.11 ± 0.22 ng/g) at SCBI. Likewise, HCC for females who whelped (*n* = 5; 3.67 ± 0.49 ng/g) was similar (*F*_1, 8.74_ = 3.54, *P* > 0.10) to females who did not whelp (*n* = 11; 2.73 ± 0.28 ng/g) at FCC.

## Discussion

The black-footed ferret recovery program has been extremely successful, producing nearly 10 000 individuals from seven genetic founders in ~35 years (Marinari, 2019). However, with out any novel genes to introduce into the population at least, naturally, *F* has continued to increase over the years from an estimated 0 to 0.1305 (Graves *et al*., 2019). As population relatedness has increased, we have observed increased infertility (declining seminal quality and whelping success) in the black-footed ferret (Santymire *et al*., 2019). The etiology of this infertility is unknown but may be related to environmental conditions since individuals living in the wild have improved semen quality and larger testes than captive males (Santymire *et al*., 2004; Santymire *et al*., 2014). Therefore, our goal was to compare GC production across *in situ* and *ex situ* black-footed ferrets, hypothesizing that chronic stress may be impacting *ex situ* reproduction. We used HCC analysis to compare different breeding facilities to a wild population of black-footed ferrets. We attempted to eliminate the issues of using hair to analyse the stress physiology by comparing HCC between populations and facilities within the same month within the season and shaving hair from the same location (ventral side of neck). This strategy was to reduce variation in the HCC due to time of year and location on the body (Carlitz *et al*., 2015; Fourie *et al*., 2016).

Because age can affect reproductive success and can influence GC production, we first chose to compare HCCs between juvenile and adult black-footed ferrets. Age has been shown to affect HCC in several species from non-human primates to cattle (reviewed in Heimbürge *et al*., 2019). We did observe higher HCCs in juveniles (~90 to 120 days of age) compared to adults that were 1 to 4 years of age. Because adolescence is a time of rapid growth, GCs may be elevated to manage the greater metabolic demands. Additionally, age class is identified by a shift in neuroendocrine functioning (Romeo, 2010, 2013). For example, studies have shown that juveniles have higher GCs than adults in the rat *Rattus rattus* (Romeo *et al*., 2004), Egyptian mongoose (*Herpestes ichneumon*; Azevedo *et al*., 2019) and mountain goats (*Oreamnos americanus*; Dulude-de Broin *et al*., 2019). Conversely, in Canadian lynx (*Lynx canadensis*; Terwissen *et al*., 2013) and brown hares (*Lepus europaeus*; Esposito *et al*., 2017), there was no significant effect of age on hair cortisol levels. The influence of life stages on glucocorticoid production is dependent on timing and species (Heimbürge *et al*., 2019). We were thus able to establish that age has a significant impact on HCCs in the black-footed ferret.

We then built a model to explain HCC variation in the adult black-footed ferrets and determined that sex, season and interactions between sex and season and *ex situ* versus *in situ* and season were significant predictors of HCC. For sex, HCCs in wild males and females reflected opposite trends depending on season. During the non-breeding season, females had significantly higher HCC than males whereas in the breeding season, males had significantly higher HCC than females. This difference may be driven by the different roles that each sex plays in reproduction. For instance, during the breeding season the males may be establishing territories since they are a polygynous species (Wingfield and Sapolsky, 2003; Livieri and Anderson, 2012). Santymire *et al*. (2020) measured FGM in male black-footed ferrets before, during and after the breeding season and observed a significant increase in FGMs during the breeding season (Santymire *et al*., 2020). In general, increases in cortisol may reflect allostatic adjustments to different energy requirements (Sapolsky *et al*., 2000; Romero *et al*., 2009), which would be especially true during the breeding season. For example, in Japanese macaques (*Macaca fuscata*), mating frequency of subordinate males was positively correlated with cortisol levels (Barrett *et al*., 2002). Wild females had higher HCC than the wild males during the non-breeding season potentially due to the increased energy demands from supporting their offspring before they reach adult size and disperse away from their mothers.

When comparing the HCC of *in situ* and *ex situ* females, during the non-breeding season, wild females had higher HCCs than females from two breeding facilities (FCC and SCBI), which were similar. Higher HCC may be due to the increased energetic demands of living in the wild. Studies have shown that moderate to higher intensity exercise causes increases in circulating cortisol levels (Hill *et al*., 2008; Gerber *et al*., 2012). Wild females live on prairies where they have access to open land and large burrows. *Ex situ* individuals live in pens supplemented with a nest box. Wild females also have to kill prey to feed their offspring, where *ex situ* females do not have to provide food to their offspring after weaning. Even during the breeding season, wild females had higher HCC than FCC females. This may be because they are experiencing different stressors, such as multiple encounters with males versus one male selected by staff to pair with the female and nutritional stress with wild females still having to hunt and kill prey. We were not able to collect hair from SCBI females in the spring because most facilities do not anesthetize females during the breeding season and samples that we used were from FCC females who were anesthetized for surgical artificial insemination. HCC for both FCC females and wild females was consistent between both seasons. Another study has observed an increase in stress hormone production (via FGMs) in FCC females as they entered the breeding season, but this study was able to collect multiple samples over consecutive weeks (Poessel *et al*., 2011).

For males, HCC during the breeding season was similar between wild and SCBI individuals but lower in FCC males and was the lowest in LZG black-footed ferrets. This could be driven by environmental conditions. Males were housed in smaller breeding cages at FCC and LZG, but black-footed ferrets at SCBI are maintained in larger pens with mulch for substrate allowing for greater intensity of activity. Previous research has determined that black-footed ferrets raised at SCBI have longer limbs, which the authors suggested was because the larger pens allow them more space to engage in physical activity (Wisely *et al*., 2005). While FCC males have more varied HCC, the majority of individuals are concentrated at lower levels than SCBI and wild males. LZG males had the lowest HCC even though their pens are identical in size to FCC pens. Here, lower HCC could be related to the management practices. For example, the building where black-footed ferrets are located at LZG has more human presence and continuous noise. FCC and SCBI are specialized breeding facilities maintained separately from other animals and away from excessive noise. LZG individuals may have become habituated to human disturbances, which might explain the significantly lower levels of HCC. Interestingly, wild male HCC increased during the breeding season compared to non-breeding season (cross-season data not available for other males). This could be related to establishing and defending territories around females. Increasing GC production during the breeding season has been observed previously in FCC males via FGM analysis (Santymire *et al*., 2020).

Overall, we did observe a relationship between HCC and reproductive success with sires at FCC having higher HCC. Our original hypothesis was that *ex situ* black-footed ferrets would have higher HCCs because it is presumed that animals in zoos have more psychological stressors such as visitor presence, the inability to disperse from social pressures, limited usable space and lack of natural diets (Morgan and Tromborg, 2007). However, zoos make great efforts to simulate the natural environment that stimulate natural behaviours for species they house. Interestingly, we found the inverse relationship with sires having higher HCC than males that did not sire that breeding season. Perhaps, low HCC is an indicator of an underlying issue of chronic stress-rated pathology where HPA axis is downregulated due to allostatic overload, which is the cumulative damage to bodily functions and health due to repeated attempts at maintaining homeostasis (reviewed in McEwen and Wingfield, 2010; Juster *et al*., 2010).

In addition to the evaluating biological relevance of the HCC for the black-footed ferrets, we should validate the biochemical analysis further. Here, we conducted the standard biochemical laboratory validations to ensure the sample’s antigens were binding to the cortisol antibody similar to the standards (the parallelism) and we confirmed that there was no interference with the sample’s hormones binding to the antibody (the percent recovery). However, future research efforts should include performing high-pressure liquid chromatography paired with simultaneous mass spectrometry (LC–MS/MS) to identify the conjugated and unconjugated immunoreactive steroids that are found in the black-footed ferret hair extracts. This will ensure that the EIA system is producing exact quantification of the steroids and that they are biologically relevant. Recent efforts have analysed the steroidal components for hair extracts from six different mammalian species including two carnivores and four omnivores using the same cortisol polyclonal antibody and HRP (Jewgenow *et al*., 2020). Interestingly, these authors determined that this cortisol EIA was over-estimating the amount of up to three times higher compared to the LC–MS/MS results; however, another EIA (cortisol-21-HS) produced 2.3 to 12 times higher HCC than this cortisol EIA. Because each analysis is species specific, it will be important to conduct the LC–MS/MS analyses on black-footed ferret HCC to validate the interpretations and to precisely identify the steroids that are being measured.

In conclusion, we demonstrated that juveniles have higher HCC than adult black-footed ferrets. In adults, HCCs were influenced by all factors in the model including sex, *in situ* versus *ex situ* and season. We determined that *ex situ* ferrets do not have higher HCC than wild individuals and high HCC were not related to poor reproductive success. In fact, it was almost the opposite relationship than what we predicted, with FCC males, who sired, with significantly higher HCC compared to the males who did not sire. This suggests that higher HCC may be important for reproduction and that low HCC may signal issues with allostatic overload or dysfunction of the HPA axis regulation. Further analysis is needed to determine the relationship between low HCC, HPA function and reproductive success in the black-footed ferret. Unfortunately, we were only able to compare the effect of location on female HCC across the seasons because we did not have any samples from *ex situ* males in the non-breeding season. In the wild, male HCC did increase in the breeding season. Santymire*et al.* (2020) observed an increase in FGMs during the breeding season in FCC males, so perhaps we would have observed a similar result in the HCC.

*Ex situ* black-footed ferret HCC did not necessarily reflect wild trends and seemed to be influenced by breeding facility. During breeding season, SCBI males exhibited similar HCCs to wild males, while FCC and LZG males had lower HCC. The low HCCs exhibited by LZG males cannot be explained by cage size alone, since FCC males live in similar sized cages, but may be related to management practices. Because the *ex situ* environments cannot completely mimic the wild environment, there may be unintended consequences (Fischer and Romero, 2019). For example, in the wild, black-footed ferrets are solitary except for a dam with her kits. Males and females typically encounter one another only during breeding season. In the *ex situ* environment, males and females live in separate cages but are housed adjacent to each other in the same room. Though not well studied, housing solitary, seasonal breeders together *ex situ* can lead to behaviour issues and sexual incompatibility, such as was the case for captive cheetahs for many years (Wielebnowski *et al*., 2002; Schulte-Hostedde and Mastromonaco, 2015; McDermott, 2019). It is a goal of zoo breeding programs to have individuals exhibit similar behaviours and physiological responses as their wild counterparts. This can impact the overall fitness of the species, which is especially critical in the case of endangered species. In the case of the *ex situ* black-footed ferret, fertility has been found to be reduced compared to wild counterparts (Santymire*et al*., 2004; Santymire *et al*., 2014). Although HCC was not found to be correlated with reproductive success, the fact that both HCC and reproductive measurements are significantly different between wild and some breeding facilities indicate that the variable environments may be increasing negative effects on fitness in the black-footed ferret, as posited by EDID theory (Cheptou and Donohue, 2011). Specifically, certain environmental conditions may increase the magnitude of inbreeding depression experienced by black-footed ferrets. While investigations are still underway to determine what specific genetic mutations the black-footed ferrets have compared with non-inbred related species, the significantly different reproductive and stress phenotypes exhibited by ferrets living under different conditions lends evidence to EDID as a culprit.

Alternatively, differential HCCs based on environmental differences can be a result of metabolic rate rather than perceived stress. As previously discussed, GCs are essential for energy mobilization, and access to larger cages and more exercise, paired with changes in seasonal breeding demands, may explain some of the differences we observed between locations (MacDougall-Shackleton *et al*., 2019). Additionally, GCs have pleiotropic effects on a large suite of genes (Xavier *et al*., 2016; MacDougall-Shackleton *et al*., 2019). It is possible that the varying levels of HCC indicate different metabolic demands and that these varying levels cause differential gene expression downstream. As aforementioned, that differential gene expression may impact fitness metrics such as reproduction.

With just an estimated 700 black-footed ferrets total living *in situ* and *ex situ*, it is important to continue to investigate the etiology of infertility. While stress does not seem to be directly impacting fitness in *ex situ* individuals, it could be contributing indirectly to causes of infertility and may be influenced by other factors, such as nutrition. For example, antioxidant deficiency can lead to oxidative stress, which has been shown to damage spermatozoa membranes and DNA (De Iuliis and Aitken, 2009; Wright *et al*., 2014). Abnormal morphology can prevent spermatozoa from reaching the ovum (Agarwal and Saleh, 2002; Pukazhenthi *et al*., 2006; Wright *et al*., 2014) and high levels of sperm DNA damage can prevent pregnancy or lead to offspring abnormalities (Ahmadi and Ng, 1999; Wright *et al*., 2014; Menezo *et al*., 2016). While oxidative stress is not measured by cortisol, diet may influence cortisol levels, and cortisol levels may thereby play a role in influencing expression in genes related to fertility (Whirledge and Cidlowski, 2017; von Krogh *et al*., 2019) that then compound with the symptoms of oxidative stress, impacting reproductive success. Studies are currently underway to understand how different diets in the black-footed ferret may promote or prevent antioxidant uptake and how this influences gene expression, oxidative stress and spermatozoa health.

The black-footed ferret program will continue to use science-based management to recover the species. Other tools of propagation are also being investigated including intra-species somatic cell nuclear transfer (Wisely, 2016). In 2021, a black-footed ferret was cloned using a cell line from 1988, marking the first ever cloning of a North American endangered species (Imbler, 2021). Additionally, we could incrementally increase gene diversity by using frozen–thawed semen from wild and/or *ex situ* founders or at least individuals that were in the population decades ago for artificial insemination as previously demonstrated (Howard *et al*., 2016). As more and more species need to be managed in *ex situ* breeding programs to avoid extinction, we can use information generated from the black-footed ferret recovery program to help inform our understanding of how to manage wild animals *ex situ*. The short generation time of black-footed ferret populations works to our advantage because we can obtain rapid results from management changes. Also, as one of the oldest and most successful *ex situ* programs, along with the California condor (*Gymnogyps californianus*) and Wyoming toad (*Anaxyrus baxteri*), we have multitudes of generations that have informed our research and that can provide information on phenotypic and genetic change in the *ex situ* breeding program that would otherwise only be available decades from now in other programs with species that might have longer generation times or a social hierarchy that prolongs breeding.
